# Eosinophilic Pleural Effusion as an Unusual Initial Manifestation of IgG4-Related Disease: A Diagnostic Challenge

**DOI:** 10.7759/cureus.91102

**Published:** 2025-08-27

**Authors:** Parinya Ruenwilai, Sarawut Kongkarnka, Athitarn Tangtermpong

**Affiliations:** 1 Division of Pulmonary, Critical Care and Allergy, Department of Internal Medicine, Faculty of Medicine, Chiang Mai University, Chiang Mai, THA; 2 Department of Pathology, Faculty of Medicine, Chiang Mai University, Chiang Mai, THA; 3 Department of Internal Medicine, Chiang Mai University Hospital, Chiang Mai, THA

**Keywords:** eosinophilic pleural effusion, exudative pleural effusion, igg4-related disease, pleural effusion, pleuroscopy

## Abstract

We report the case of a 79-year-old male who presented with progressive dyspnea over the course of one month. Chest radiography revealed a left-sided pleural effusion. Pleural fluid analysis demonstrated an exudative profile with eosinophil-predominant cytology. Medical thoracoscopy identified multiple hyperpigmented spots on the anterior parietal pleura. Histopathological examination of pleural biopsy specimens confirmed the diagnosis of IgG4-related disease (IgG4-RD). The pleural effusion resolved completely following corticosteroid therapy.

## Introduction

Eosinophilic pleural effusion is defined by the presence of ≥10% eosinophils in the pleural fluid and accounts for approximately 10% of exudative pleural effusions. According to previous studies, the most common cause of eosinophilic pleural effusion is malignancy (52.94%), followed by idiopathic causes (14.71%), parasitic infections (8.82%), parapneumonic effusion (8.82%), and other causes (14.71%). However, eosinophilic pleural effusion can also occur in association with chronic systemic inflammatory diseases such as rheumatoid arthritis (RA), systemic lupus erythematosus (SLE), and eosinophilic granulomatosis with polyangiitis (EGPA) [1]. Here, we report a case of eosinophilic pleural effusion as the initial presentation of IgG4-related disease (IgG4-RD), a rare and underrecognized etiology.

## Case presentation

**Figure 1:**
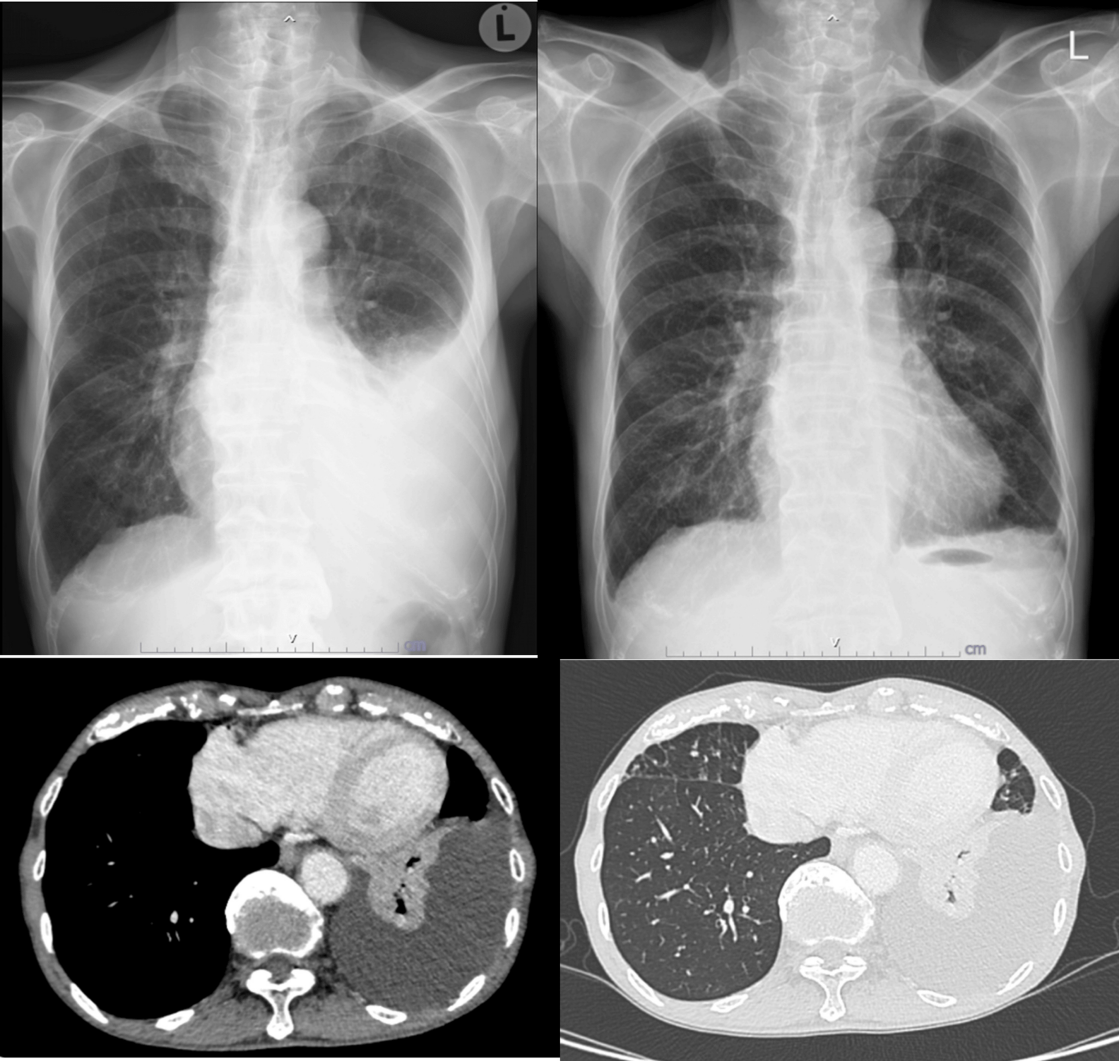
Left: Chest X-ray shows left pleural effusion. Right: Chest X-ray after two weeks of prednisolone treatment, demonstrating resolution of the effusion. The bottom row shows CT images revealing left pleural effusion without pleural thickening or significant mediastinal lymphadenopathy.

A 79-year-old male with a history of chronic obstructive pulmonary disease (COPD), bronchiectasis, and non-valvular atrial fibrillation presented with progressive dyspnea persisting for one month, without orthopnea, paroxysmal nocturnal dyspnea, fever, or weight loss. He had a history of chronic tobacco and alcohol use. Chest radiography revealed a left-sided pleural effusion (Figure 1), which was confirmed by computed tomography (CT) along with compressive atelectasis, but no discernible lung or pleural masses (Figure 1).

The pleural fluid analysis from the initial thoracentesis revealed an exudative effusion characterized by a total white blood cell (WBC) count of 2791 cells/cu.mm, with a differential count showing 24.2% neutrophils, 35.8% eosinophils, and 40% lymphocytes. The pleural fluid protein level was 3.1 g/dL, and the lactate dehydrogenase (LDH) was 250 U/L, consistent with an exudative process (serum LDH: 207 U/L). Cytological analysis confirmed eosinophil-rich fluid. Notably, the patient's serum IgG was elevated at 2010 mg/dL, with a markedly elevated IgG4 level of 939 mg/dL (Table 1).

**Table 1:** Pleural fluid analysis table. LDH: lactate dehydrogenase, WBC: white blood cell.

Parameter	Patient value	Reference range
Appearance	Clear	Clear
Total WBC	2791 cells/mm³	<1000 (transudate)
Neutrophils (%)	24.2%	<25%
Eosinophils (%)	35.8%	<10%
Lymphocytes (%)	40%	<75%
Protein	3.1 g/dL	>2.9 g/dL (exudate)
LDH	250 U/L	>2/3 serum LDH (exudate)
Serum LDH	207 U/L	

**Figure 2:**
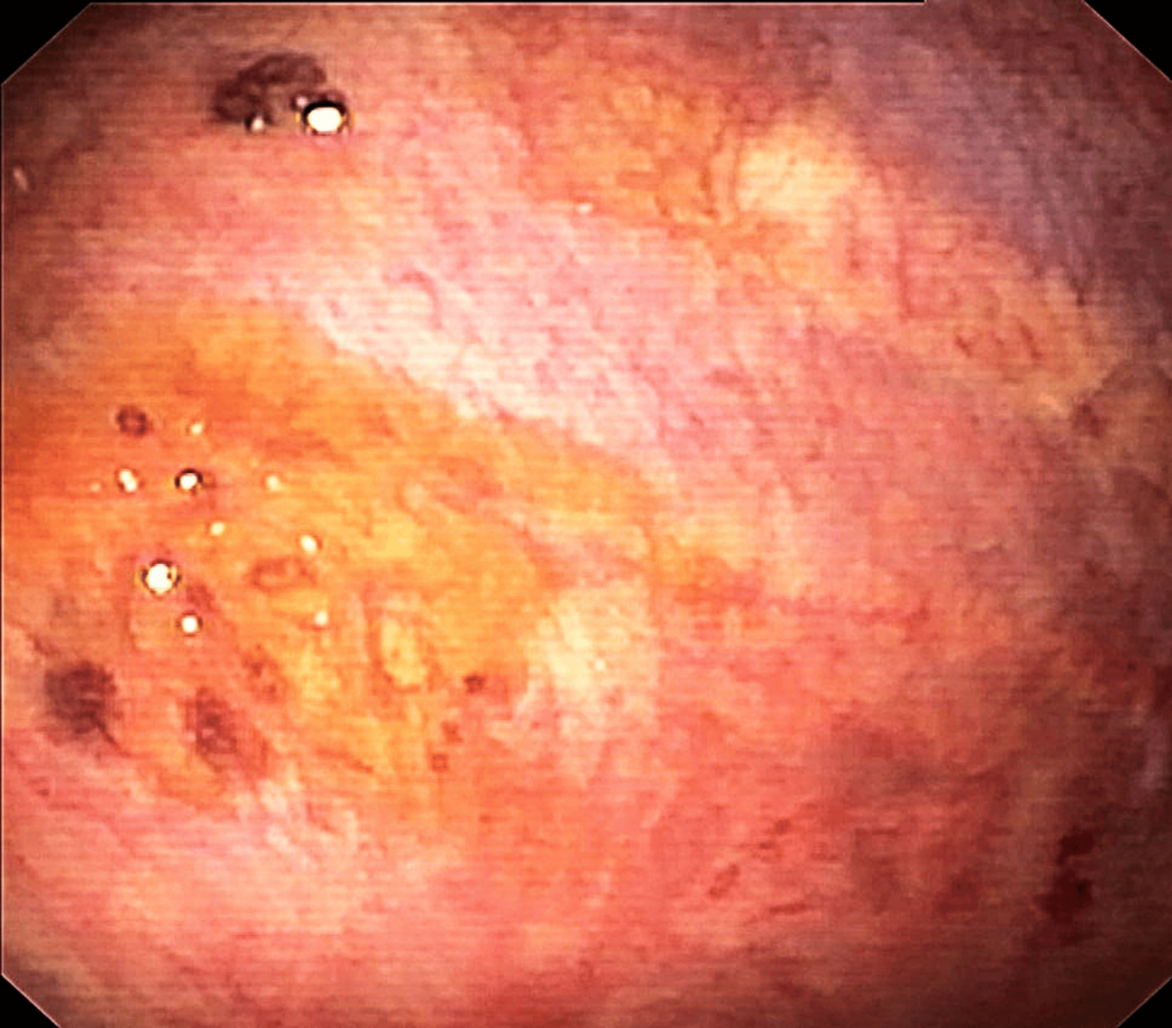
Medical thoracoscopy revealed multiple hyperpigmented spots and yellowish plaques on the anterior parietal pleura.

**Figure 3:**
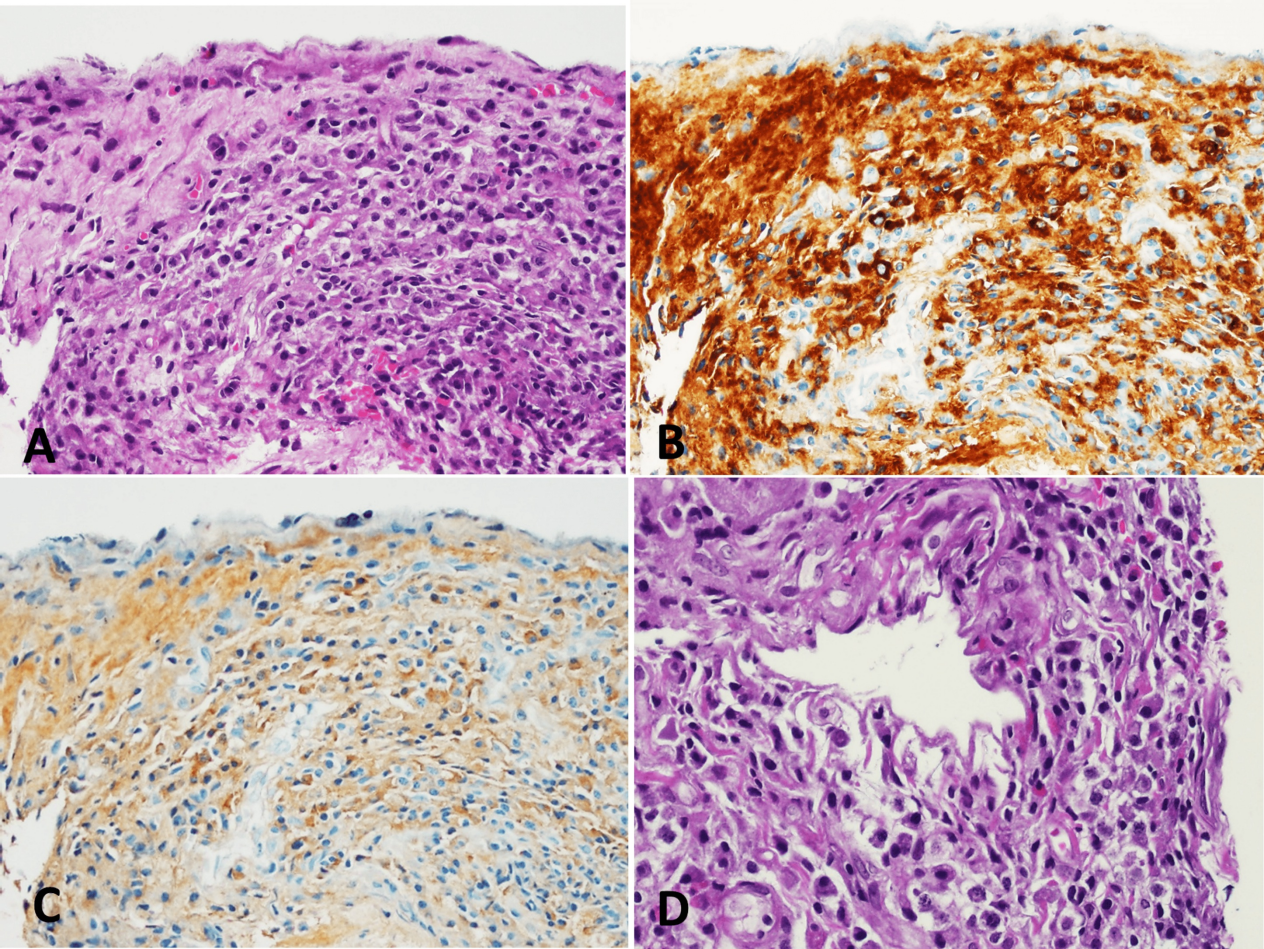
(A) Pleural tissue shows dense lymphoplasmacytic infiltrate without fibrosis (400x magnification). (B) and (C) Sections B and C represent immunohistochemical stains for IgG4 and IgG, respectively. These stains demonstrated a density of 155 IgG4-positive plasma cells per square millimeter, yielding an IgG4:IgG-positive plasma cell ratio of 72.61% (at 400x magnification). (D) Perivascular lymphoplasmacytic infiltration is present, but typical obliterative phlebitis is not observed. Additionally, there is increased eosinophilic infiltration, reaching up to 12 eosinophils per high-power field (400x magnification) in hotspot areas.

Medical thoracoscopy revealed multiple hyperpigmented spots and yellowish plaques on the anterior parietal pleura (Figure 2). Pleural biopsy showed dense lymphoplasmacytic infiltrates and fibrosis, with 155 IgG4-positive cells/mm² and an IgG4/IgG plasma cell ratio of 72.6%, confirming IgG4-related disease (Figure 3). The patient was started on oral prednisolone (0.6 mg/kg/day) for two weeks, leading to marked radiographic improvement (Figure 1).

## Discussion

IgG4-related disease (IgG4-RD) is an immune-mediated inflammatory disorder characterized by fibroinflammatory lesions that can affect multiple organs. It predominantly affects males, particularly between the fifth and seventh decades of life, and has been associated with exposure to tobacco and asbestos. The clinical presentation varies depending on the organs involved and is typically insidious in onset, often lacking systemic symptoms such as fever for months or even years [2].

The most commonly affected organs in IgG4-related disease (IgG4-RD) include the lacrimal glands, major salivary glands, pancreas, and retroperitoneum. Less frequently involved sites include the lungs, kidneys, bile ducts, and orbits, while rare involvement has been reported in the pachymeninges, thyroid, and aorta. Diagnosis of IgG4-RD requires a combination of clinical and radiologic findings, elevated serum IgG4 levels (>135 mg/dL), and characteristic histopathological features. A favorable response to corticosteroid therapy is also a hallmark of the disease [3].

Pleural involvement has been observed in approximately 16.1% of IgG4-RD cases [1]. It can manifest as a pleural mass, pleuritis with fibrosis, pleural effusion, pleural nodules on the visceral or parietal pleura, or focal/diffuse pleural thickening with or without parenchymal involvement [4]. IgG4-RD with pleural involvement has been reported to show pleural hyperemia, fibrotic plaques, and multiple pleural nodules on thoracoscopic examination [5,6]. 

Pleural effusion occurs in about 4.6% of IgG4-RD cases. These effusions are typically exudative, rich in lymphocytes and plasma cells, and often present without involvement of other organs. Additionally, there has been a case report of IgG4-RD presenting with chylothorax, with an unknown mechanism [7]. However, exudative pleural effusion due to IgG4-RD is difficult to distinguish from sarcoidosis, SLE, RA, or multicentric Castleman's disease (MCD) [1]. Kasashima et al. found that pleural fluid in IgG4-RD cases had a higher percentage of eosinophils in fluid cell counts, more eosinophils per high-power field in cytology, and greater eosinophilic infiltration in histology, compared to pleural effusion in non-IgG4-RD cases [8].

Pleural involvement in IgG4-related disease (IgG4-RD) may present in isolation or alongside pulmonary and extrapulmonary manifestations. Although increasingly recognized, it remains underdiagnosed. Enhancing clinician awareness across specialties is essential, as IgG4-RD may underlie a subset of pleural effusions currently labeled as idiopathic. In cases of unexplained pleural effusion, particularly those with histological evidence of lymphoplasmacytic infiltration, IgG4-RD should be considered in the differential diagnosis. Multidisciplinary collaboration between clinicians, radiologists, and pathologists is vital to ensure accurate diagnosis and timely treatment [9].

## Conclusions

In this report, we describe the stepwise diagnostic approach in a patient who presented with an eosinophilic exudative pleural effusion. After excluding infectious and malignant causes, attention was directed toward immune-mediated conditions. The diagnosis of IgG4-RD was ultimately confirmed through histopathological examination of a pleural biopsy specimen. Further research in larger patient cohorts is warranted to better define the prevalence, natural history, and treatment outcomes of pleural involvement in IgG4-RD.
